# Genetic counselling implementation in dilated cardiomyopathy: A clinical consensus statement of the Heart Failure Association of the ESC, the ESC Working Group on Myocardial and Pericardial Diseases, and the ESC Council on Cardiovascular Genomics

**DOI:** 10.1093/eurheartj/ehag159

**Published:** 2026-03-20

**Authors:** Job A J Verdonschot, Karin Y van Spaendonck-Zwarts, Debby M E I Hellebrekers, Folkert W Asselbergs, Elijah R Behr, Philippe Charron, Dana Dawson, Pablo Garcia-Pavia, Kristina H Haugaa, Ruxandra Jurcut, Petr Kuchynka, Luis R Lopes, Andrea Mazzanti, Marco Metra, Lorenzo Monserrat, Juan Pablo Kaski, Antonis Pantazis, Sanjay K Prasad, Giuseppe Rosano, Petar M Seferovic, Mary N Sheppard, Gianfranco Sinagra, Maria Teresa Tome Esteban, Stephane R B Heymans, J Peter van Tintelen

**Affiliations:** Department of Clinical Genetics, Maastricht University Medical Center+, P. Debyelaan 25, Maastricht 6229HX, The Netherlands; Department of Cardiology, Cardiovascular Research Institute Maastricht, Maastricht University, Maastricht, The Netherlands; Department of Clinical Genetics, Leiden University Medical Center, Leiden, The Netherlands; Department of Genetics, University Medical Center Groningen, Groningen, The Netherlands; Department of Clinical Genetics, Maastricht University Medical Center+, P. Debyelaan 25, Maastricht 6229HX, The Netherlands; Department of Cardiology, Amsterdam Cardiovascular Sciences, Amsterdam University Medical Centre, University of Amsterdam, Amsterdam, The Netherlands; Institute of Health Informatics, University College London, London, UK; The National Institute for Health Research University College London Hospitals Biomedical Research Centre, University College London, London, UK; Cardiovascular and Genomics Research Institute, City St. George’s, University of London, London, UK; Cardiovascular Clinical Academic Group, St. George’s University Hospitals NHS Foundation Trust, London, UK; Filière Nationale de Santé CARDIOGEN, Paris, France; Genetics and Cardiology Departments, APHP, Sorbonne Université, INSERM 1166, Institute of Cardiology and ICAN Institute for Cardiometabolism and Nutrition, Pitié-Salpêtrière Hospital, Paris, France; Aberdeen Cardiovascular and Diabetes Centre, University of Aberdeen, Aberdeen, UK; Heart Failure and Inherited Cardiac Diseases Unit, Department of Cardiology, Hospital Universitario Puerta de Hierro IDIPHISA, Madrid, Spain; CIBER Cardiovascular Instituto de Salud Carlos III, Madrid, Spain; Centro Nacional de Investigaciones Cardiovasculares (CNIC), Madrid, Spain; Department of Cardiology, Karolinska University Hospital, Stockholm, Sweden; ProCardio Center, Department of Cardiology, Oslo University Hospital, Oslo, Norway; Expert Center for Genetic Cardiovascular Diseases, Emergency Institute for Cardiovascular Diseases ‘Prof. Dr. C.C. Iliescu’, University of Medicine and Pharmacy ‘Carol Davila’, Bucharest, Romania; 2nd Department of Medicine, Department of Cardiovascular Medicine, First Faculty of Medicine, Charles University and General University Hospital, Prague, Czech Republic; Barts Heart Centre, St Bartholomew’s Hospital, London, UK; Institute of Cardiovascular Science, University College London, London, UK; Molecular Cardiology Unit, IRCCS Istituti Clinici Scientifici Maugeri, Pavia, Italy; Department of Molecular Medicine, University of Pavia, Pavia, Italy; Cardiology, ASST Spedali Civili di Brescia, Department of Medical and Surgical Specialties, Radiological Sciences, and Public Health, University of Brescia, Brescia, Italy; Medical Department, Dilemma Solutions, A Coruña, Spain; Centre for Paediatric Inherited and Rare Cardiovascular Disease, University College London, Institute of Cardiovascular Science, London, UK; Centre for Inherited Cardiovascular Diseases, Great Ormond Street Hospital, London, UK; Department of Cardiology, Royal Brompton and Harefield Hospitals, London, UK; Department of Cardiology, Royal Brompton and Harefield Hospitals, London, UK; Cardiovascular Clinical Academic Group, St. George’s University Hospitals NHS Foundation Trust, London, UK; Department of Cardiology, Serbian Academy of Sciences and Arts and Faculty of Medicine, University of Belgrade, Belgrade, Serbia; CRY Cardiovascular Pathology Unit, Cardiovascular and Genetic Research Institute, St.George’s, University of London, London, UK; Cardiothoracovascular Department, Azienda Sanitaria Universitaria Giuliano Isontina, Center for Cardiomyopathies, University of Trieste, Trieste, Italy; Cardiovascular and Genomics Research Institute, City St. George’s, University of London, London, UK; Cardiovascular Clinical Academic Group, St. George’s University Hospitals NHS Foundation Trust, London, UK; Department of Cardiology, Cardiovascular Research Institute Maastricht, Maastricht University, Maastricht, The Netherlands; Department of Cardiovascular Sciences, Centre for Molecular and Vascular Biology, University of Leuven, Leuven, Belgium; Department of Genetics, University Medical Center Utrecht, Heidelberglaan 100, Utrecht 3584CX, The Netherlands

**Keywords:** Dilated cardiomyopathy, Genetic testing, Pathogenic variant

## Abstract

Genetic testing has become an integral part of the diagnostic workup of patients with dilated cardiomyopathy (DCM). While the initial goal of genetic testing was to identify family members at risk, recent advances have now extended their relevance to clinical decision-making. Our knowledge of the genetic architecture of DCM has expanded significantly, promoting a shift from the monogenic dogma towards a broader polygenic spectrum. However, current genetic testing strategies still primarily rely on the model of monogenic inheritance with an incomplete penetrance. Large studies have shown a yield varying from 8% to 36% of genetic testing in patients with DCM, depending on aetiology or family history. Genetic testing is generally warranted for every patient with DCM where genetic results could have an impact on risk stratification, the prognosis or the treatment of the patient, or its family members with an opportunity for reassurance or early disease detection. There are various strategies for genetic testing including broad multigene panels, or more targeted panels limited to specific disease-associated genes. Identified variants are classified by genetic laboratories, where pathogenic or likely pathogenic variants often have actionable clinical implications. It is crucial to interpret these variants in the context of the individual patient considering the phenotype and other contributing factors. When the genetic results are consistent with the patients’ broader phenotype, potential clinical implications may include decision for device therapy, recommendations for family screening, and reproductive options. A comprehensive approach to integrate genetic testing in the clinical care of patients with DCM is proposed.

## Graphical Abstract

**Graphical Abstract:**
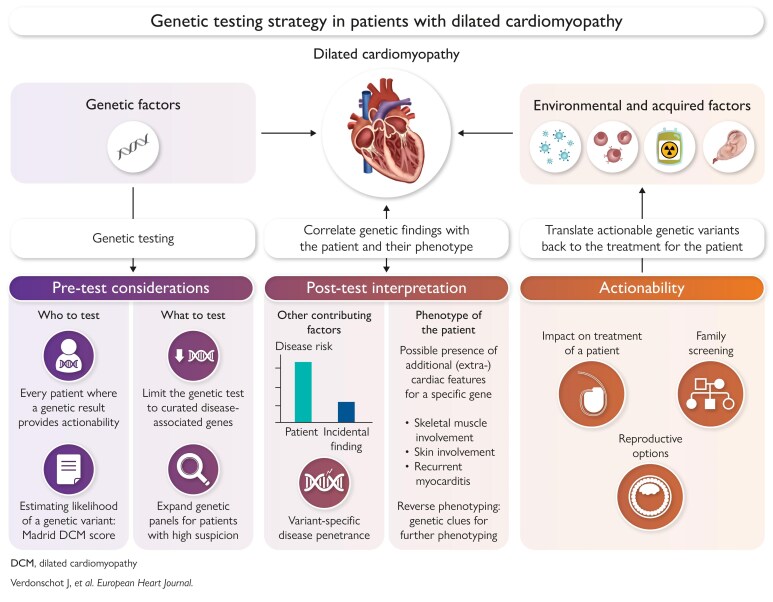
Graphical abstract. Genetic testing is indicated for every patient with dilated cardiomyopathy (DCM) if a positive genetic results would provide actionability. Before genetic testing is requested, the choice of panel (size and gene content) should be based on the clinical context of the patient. When receiving a genetic result, it is important to also interpret these results in the clinical context of the patient, and whether there are specific considerations for risk stratification and phenotyping. A genetic results can impact clinical decision-making such as ICD implantation, it will initiate family screening and provide reproductive options.

## Introduction

Dilated cardiomyopathy (DCM) is a heterogeneous disease that can be caused by both environmental and genetic triggers and combinations thereof. Recent data have shown that an apparently acquired aetiology for DCM does not exclude the presence of an underlying disease-associated genetic variant, emphasizing the importance of considering genetic testing in all patients with DCM. Moreover, our increasing understanding of the genetic architecture of DCM and rapid evolution of genetic testing technologies continue to influence clinical practice. Therefore, this clinical consensus statement aims (i) to describe the current knowledge of the genetic basis of DCM and (ii) to provide a roadmap on genetic testing and the interpretation of results in the context of individual patients and their families. Management and treatment of patients with DCM are beyond the scope of this document as this is covered in existing European Society of Cardiology (ESC) guidelines.^1,2^ In relation to the 2023 ESC guidelines on cardiomyopathies, this document expands on the complex gene–environmental interactions of DCM, the concept of genetic testing strategies, and adds guidance on the interpretation of genetic testing results in the context of the phenotype and other contributing factors of the patient with DCM (*Graphical Abstract*).

## Genetic aetiology of dilated cardiomyopathy

DCM is familial [defined as two or more individuals with DCM (first- or second-degree relatives), or if there is an index patient who fulfils the diagnostic criteria for DCM and a first-degree relative with autopsy-proven DCM who experienced sudden death before the age of 50^3,4^] in up to 30% of cases,^5^ suggesting a strong genetic component of the disease. A pathogenic or likely pathogenic (P/LP) variant is found in 19% of patients with DCM increasing to 55% in patients with a familial form of DCM.^5,6^ Genetic variants in genes encoding components of the cardiac sarcomere, structural proteins, nuclear envelope, energy metabolism, and calcium handling are known to underlie primary forms of the disease. Most genes associated with genetic DCM follow an autosomal dominant inheritance mode, while autosomal recessive, X-linked, or mitochondrial inheritance have also been reported. Also, besides isolated DCM, there can be overlap with neuromuscular diseases, inflammatory conditions, and apparent primary arrhythmic presentations.

Technological advancements in next-generation sequencing over the last two decades have enhanced genetic testing of patients with DCM and increased the number of genes tested. This has resulted in rapid identification of new putative DCM-causing genes and expansion of routine diagnostic gene panels. For clinical purposes, it is crucial that the genes included in the panels have an established gene–disease relationship in order to impact clinical decision-making. Otherwise, P/LP variants may be identified in genes that are not definitively associated with the disease, and misinterpretation in relation to the clinical diagnosis can potentially result in false-positive results. This may lead to unwarranted cascade genetic testing, unnecessary stress for families, and wasted healthcare resources. Critical (re-)evaluation of DCM gene–disease relationships is therefore warranted. The other way around is also possible, P/LP variants detected in DCM-associated genes in patients without a DCM phenotype, so-called genetic incidental findings. Interpretation of these genetic findings should be performed with caution, as the penetrance of these variants can significantly differ from P/LP variants detected in patients with DCM (also further discussed in the paragraph on *the interpretation of genetic results*).

One of the activities of PanelApp and ClinGen, an NIH-funded initiative, aims to validate gene–disease relationships. Their efforts for DCM have been previously published and are regularly being updated.^7,8^ The ClinGen DCM gene curation expert panel performed a systematic curation of evidence for 51 DCM-associated genes to establish the relationship with DCM, using published clinical genetic and experimental laboratory evidence. This resulted in 11 definitive-evidence (*BAG3*, *DES*, *FLNC*, *LMNA*, *MYH7*, *PLN*, *RBM20*, *SCN5A*, *TNNC1*, *TNNT2*, *TTN*), 1 strong-evidence (*DSP*), and 7 moderate-evidence (*ACTC1*, *ACTN2*, *JPH2*, *NEXN*, *TNNI3*, *TPM1*, *VCL*) genes for monogenic DCM without any additional features (*Table 1*). These 19 high-evidence genes are advised for routine clinical genetic testing of patients with DCM. Several of these genes have also been curated as high-evidence for other cardiac phenotypes, like *SCN5A* for Brugada syndrome,^11^  *DES* and *DSP* for arrhythmogenic right ventricular cardiomyopathy,^9^ and *ACTC1*, *MYH7*, *TNNI3*, *TNNT2*, and *TPM1* for hypertrophic cardiomyopathy.^10^ According to the European Molecular Genetics Quality Network (EMQN) recommendations for genetic testing in inherited cardiomyopathies and arrhythmias,^14^ the *DMD* gene could be added to the list of robust DCM genes, on the basis of existing evidence that *DMD* variants are causal of X-linked isolated DCM.^13^ Although ongoing gene curation efforts will change the content of future diagnostic DCM gene panels, currently these 20 genes are considered as the core genes for DCM.

**Table 1:** DCM disease genes advised for routine clinical genetic testing

Gene	Pattern of inheritance	Level of evidence^7^	Strong evidence to be associated with other cardiac diseases
*BAG3*	Ad	Definitive	
*DES*	Ad	Definitive	ARVC^9^
*DSP*	Ad	Strong	ARVC^9^
*FLNC*	Ad	Definitive	
*LMNA*	Ad	Definitive	
*MYH7*	Ad	Definitive	HCM^10^
*PLN*	Ad	Definitive	ARVC,^9^ intrinsic CM
*RBM20*	Ad	Definitive	
*SCN5A*	Ad	Definitive	Brugada syndrome,^11^ long QT syndrome^12^
*TNNC1*	Ad	Definitive	HCM^10^
*TNNT2*	Ad	Definitive	HCM^10^
*TTN*	Ad	Definitive	
*ACTC1*	Ad	Moderate	HCM^10^
*ACTN2*	Ad	Moderate	HCM^10^
*JPH2*	Ad	Moderate	HCM^10^
*NEXN*	Ad	Moderate	
*TNNI3*	Ad	Moderate	HCM^10^
*TPM1*	Ad	Moderate	HCM^10^
*VCL*	Ad	Moderate	
*DMD*	XL	Other^13^	

Ad, autosomal dominant; ARVC, arrhythmogenic right ventricular cardiomyopathy; CM, cardiomyopathy; HCM, hypertrophic cardiomyopathy; XL, X-linked.

In addition to DCM being an isolated (familial) cardiac trait, it can also be a part of a broader phenotypic spectrum that may include dysmorphic features (often in the context of syndromes) such as Alstrom syndrome, along with skeletal myopathies or other features including developmental delay and hearing loss (e.g. in the context of mitochondrial and metabolic diseases). Disease onset in childhood can be an additional ‘red flag’ to consider rarer genetic causes.^15,16^ In these cases, expansion of the number of genes tested beyond the core genes summarized in *Table 1* is advised, because other aetiologies (e.g. metabolic diseases and syndromes) may underlie the disease.

**Figure 1:**
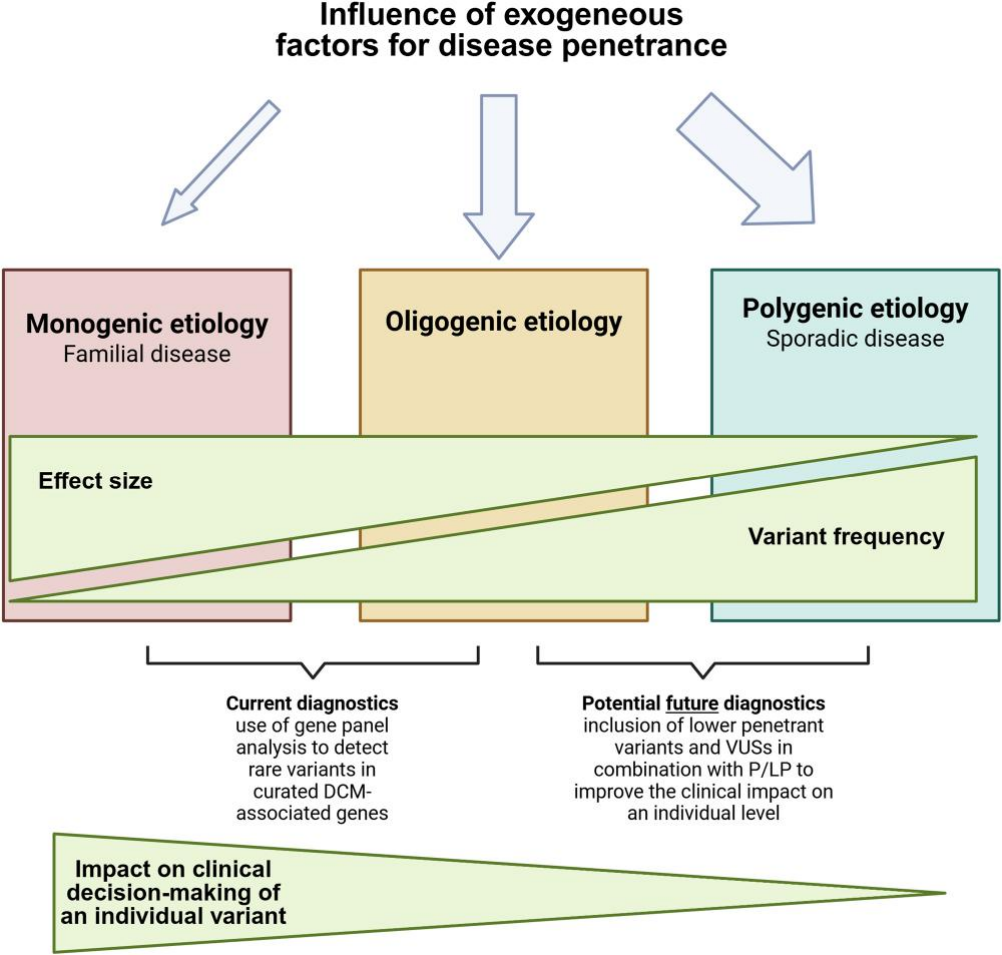
Genetic aetiology of dilated cardiomyopathy (DCM). A monogenic aetiology suggests that (often) one rare variant with a large effect size is sufficient to cause DCM, while a polygenic aetiology refers to a combination of multiple common variants that all have a small contribution to DCM risk. The current diagnostic framework in clinical practice aims to identify monogenic aetiologies that can form the basis for clinical decision-making. P/LP, pathogenic/likely pathogenic; VUS, variant of uncertain significance

In the current era, the paradigm of DCM as a purely monogenic disease, where a P/LP variant in a single gene is considered sufficient to cause disease, is increasingly being challenged. Instead, disease expression is now thought to result from an interplay between rare variants with large effect sizes, variants with moderate or small effect sizes (which may be very rare or relatively common), and/or exogenous factors (*Figure 1*). Current clinical practice focuses on identifying rare P/LP variants that can have clinical implications for the patient by using targeted gene panels. This does not preclude the possibility that some variants of uncertain significance (VUSs) may actually be P/LP, highlighting the importance of regular variant reclassification. In contrast, polygenic risk scores containing common polymorphisms (i.e. genetic variants with a relatively high frequency in the population) have been identified to be associated with DCM.^17–19^ The addition of many of such adverse, relatively common, variants to the rare P/LP variants might explain why a patient develops disease and provide hints of the pathophysiology. Although these common variants currently have no clinical implications, they hold potentially great promise for future diagnostics in patients with DCM.

## Gene–environmental interactions in dilated cardiomyopathy

With current diagnostic possibilities, ∼6% of patients with DCM have a combined genetic and non-genetic aetiology.^5^ The clinical presentation and outcome of DCM are often determined by the co-existence of genetic factors with environmental and acquired factors.^20–23^ The impact of acquired factors on disease penetrance differs per genotype.^24^ For this reason, most clinical knowledge is available on high penetrant genotypes that generally do not require environmental factors to develop a phenotype (e.g. *LMNA*). In contrast, carriers of a truncating variant (tv) in *TTN*, the most prevalent genetic aetiology of DCM, often do not develop a phenotype in the absence of additional (environmental) triggers.^5,25–27^ Examples of triggers contributing to the development of DCM in *TTNtv* carriers include:

- *Excessive alcohol intake*: *TTNtv* forms a genetic predisposition for alcohol-induced cardiomyopathy and is associated with a lower left ventricular ejection fraction (LVEF) in patients with DCM who consume alcohol above recommended levels.^28^
- *Cardiotoxic chemotherapy*: *TTNtv* increases the risk of chemotherapy-induced cardiomyopathy in children and adults and adverse cardiac events in adults.^29^ Consistent with human data, anthracycline-treated *TTNtv* mice show sustained contractile dysfunction unlike wild-type. Genotype, along with cumulative cardiotoxic chemotherapy dosage and traditional cardiovascular risk factors, improves the identification of patients with cancer at higher risk of developing DCM.
- *Myocarditis*: *TTNtv* is prevalent in patients with myocarditis and a reduced LVEF and in biopsy-proven paediatric myocarditis.^2,30,31^ Paediatric patients with myocarditis who develop DCM and have a *TTNtv* are characterized by early-onset heart failure and poor outcome. While *TTNtv* often requires environmental triggers such as viral myocarditis to unmask disease, P/LP *DSP* variants are increasingly recognized to cause myocarditis-like episodes themselves, independent of infection.^32^ This further illustrates the continuum between genetic and inflammatory cardiomyopathies.
- *Pregnancy*: women with peripartum cardiomyopathy (PPCM) have a similar prevalence of *TTNtv* compared with patients with DCM, establishing a comparable genetic predisposition.^33,34^ This supports the notion that PPCM can be an initial manifestation of DCM in a family with a *TTNtv*.^2^
- *Systemic immune-mediated diseases:* ∼8%–11% of patients with DCM due to an underlying systemic immune-mediated disease (e.g. psoriasis and Sjogren disease) also have a *TTNtv*.^35^ Those patients also have a significantly lower LVEF at first presentation compared with patients with DCM without the combination.

The presence of rare P/LP variants in DCM-associated genes also impacts left ventricular reverse remodelling and outcome in patients with stable coronary artery disease.^36^ Genetic testing may be appropriate in patients with coronary artery disease and disproportionate left ventricular systolic dysfunction and may highlight a subgroup of individuals that would benefit from enhanced medical surveillance.

Overall, these studies support a model in which environmental factors interact with the genotype to determine the cardiac phenotype, playing an important role in unveiling a phenotype in carriers of low and high penetrant gene variants. This further highlights that the finding of a non-genetic aetiology does not exclude a genetic aetiology and supports the recommendation that genetic testing may be appropriate in patients with DCM due to an environmental factor.^2^ Currently, genetic testing in non-affected individuals at risk due to exposure to an environmental factor (e.g. patients undergoing cardiotoxic chemotherapy) is not advised, but at least family history should be taken.^2^ Future studies should investigate the potential benefit of this ‘reversed’ approach.

## The role of genetic testing and counselling in the diagnostic workup of a patient with dilated cardiomyopathy

The first step towards genetic testing is appropriate counselling of the patient to help patients understand the consequences of a potential genetic factor contributing to their DCM. It should be provided by appropriately trained healthcare professionals. Genetic counselling covers a broad range of topics, such as medical, genetic, and psychosocial factors. It includes discussing inheritance risks, providing educational information on clinical aspects including regular evaluations, offering pre- and post-genetic test counselling, and reviewing and clarifying variant classifications.^2,37^ Psychosocial support should be discussed and offered, as adapting to a new diagnosis—especially one with genetic and potentially life-threatening implications—can have a profound psychosocial impact. This impact may stem in part from experiences of grief or fear, such as those following resuscitation, implantable cardioverter-defibrillator (ICD) implantation, or sudden cardiac death in a family member. Attention to the psychological support needs of patients is therefore paramount. The supportive role of the growing patient advocacy groups and networks should be acknowledged, and healthcare professionals should be familiar with the local and (inter-)national groups and networks.

### Who to test

The 2023 ESC guidelines on the management of cardiomyopathies recommend performing genetic testing in every patient with DCM when it enables diagnosis, prognostication, therapeutic stratification, or reproductive management of the patient or where it enables cascade genetic evaluation of their relatives who would otherwise be enrolled into long-term surveillance.^2^ The a priori chance of finding a genetic substrate differs per individual patient. The genetic yield is highest in individuals without any other aetiologies that could contribute to disease, especially in those with familial disease (*Table 2*). In the presence of other acquired aetiologies, the a priori chance of finding a genetic substrate is slightly lower, but still considerable (8%–18%) (*Table 2*). This underscores the importance of not excluding these patients from genetic testing, as the absence of a family history or the presence of other aetiologies does not rule out a potential genetic aetiology.

**Table 2:** Yield of genetic testing stratified on different aetiologies of dilated cardiomyopathy

Clinical factor	Prevalence of rare variants in DCM-associated genes	A priori yield of a P/LP variant^a^	Reference
Family history-based			
Idiopathic	19%–22%	Reasonable	^5,6^
Familial disease	36%–55%	High-yield	
Aetiology-based			
Excessive alcohol consumption	8%–18%	Low-yield to reasonable	^28–30,33,35^
Peripartum cardiomyopathy			
Cardiotoxic chemotherapy			
Cardiac inflammation/myocarditis			
Systemic immune-mediated disease			

DCM, dilated cardiomyopathy; P/LP, pathogenic/likely pathogenic.

^a^In resource-limited settings, genetic testing could be restricted to those patients with the highest a priori yield for a P/LP variant. Data are available on the yield for some individual clinical factors, but patients often present with multiple factors. The Madrid DCM Genotype Score (https://madriddcmscore.com/) is the only available model combining family history, skeletal muscle disease, left bundle branch block, low QRS voltage, and hypertension. However, data on yield for several factors remain unknown (e.g. age categories and borderline phenotypes).

Careful phenotyping of a patient can help identify red flags that indicate which patients are mostly likely to benefit from genetic testing in settings where routine genetic testing is not available or when there are resource constraints (i.e. to select those patients with the highest probability of having a genetic aetiology).^38^ The Madrid DCM Genotype Score consists of five clinical variables (family history of DCM, skeletal muscle disease, absence of left bundle branch block, low QRS voltage in the limb leads, and absence of hypertension) that calculate the a priori probability of the presence of a P/LP variant.^39^ Additionally, the presence of a high arrhythmic burden at baseline also provides a clue for an underlying genetic aetiology.^5^ Further refinement of the clinical characteristics of DCM patients with a P/LP variant is important to help implement genetic testing in resource-limited settings (e.g. genetic yield per age category and combinations of factors) (*Table 2*).

### What to test

The advantages and limitations of different strategies of DNA sequencing (e.g. single gene vs whole exome or whole genome sequencing) have been described in detail in a previous clinical consensus statement.^40^ Currently, some genetic laboratories still perform multigene panel testing with over 400 genes associated with cardiac diseases included. Despite the large number of genes on panels, only a minority will have clinical implications for patients with DCM, as only a few genes have an established gene–disease association.^7,41^ Consistent with the limited number of high-evidence DCM genes, studies have shown that the additional diagnostic yield of a large panel of genes associated with cardiac disease as compared with a small panel of high-evidence DCM genes is negligible, only leading to the identification of more VUSs without a corresponding significant increase in causal P/LP variants.^42,43^ Expanding diagnostic gene panels can lead to a reduction in the clinical utility and cost-effectiveness of genetic testing, while increasing the risk of misdiagnosis.

**Figure 2:**
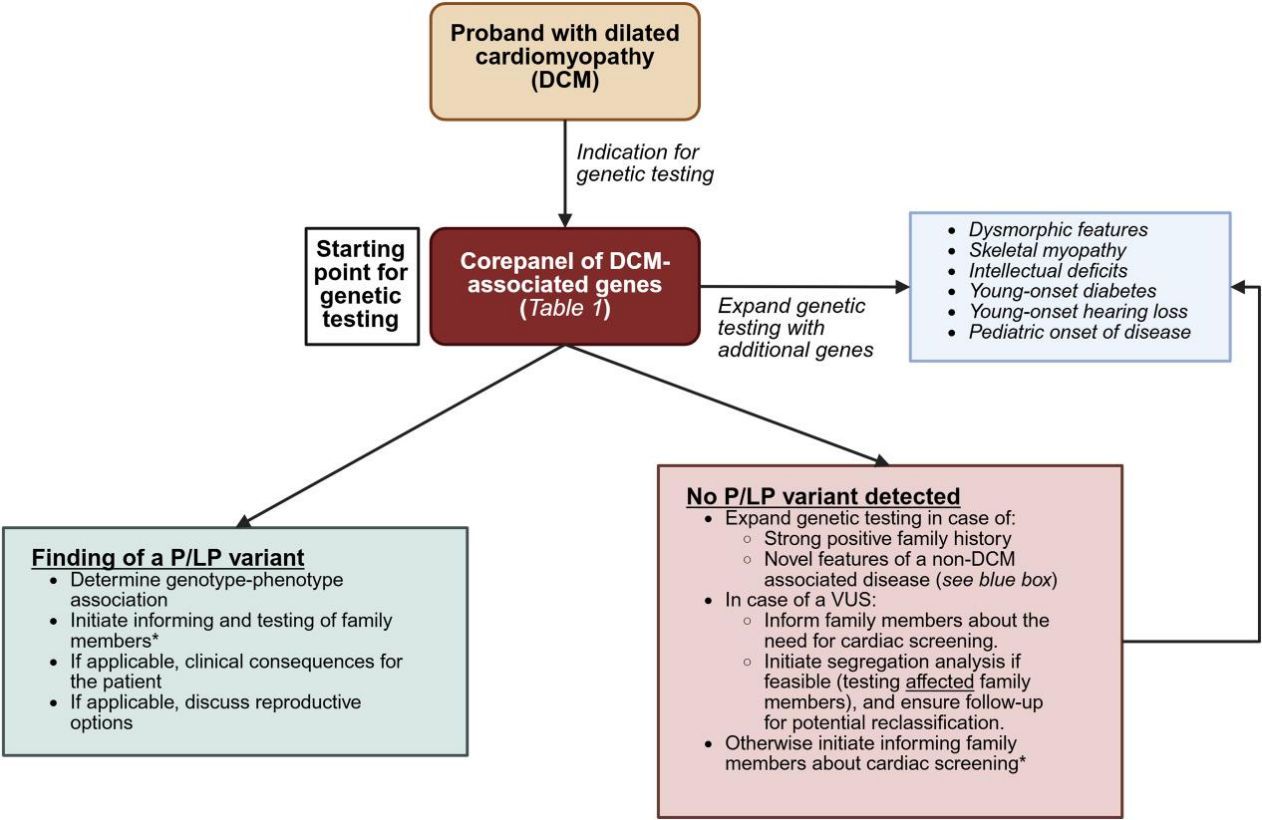
Genetic testing strategy in patients with dilated cardiomyopathy (DCM). A targeted panel of DCM-associated genes is preferred in the absence of dysmorphic features, skeletal myopathy, intellectual deficits, young-onset diabetes or hearing loss, or a paediatric onset of disease. When no pathogenic/likely pathogenic (P/LP) variant is detected, genetic testing can be expanded after considering the clinical and familial situation of the patient. In case of a variant of uncertain significance (VUS), only affected family members can be tested for the familial variant to pursue reclassification of the variant. Although some VUSs might be suspicious, current clinical decision-making is only based on P/LP variants. *See the clinical consensus statement on family screening for further details^4^

However, there are multiple approaches to genetic testing for patients with DCM, ranging from broad multigene panel analyses (which may also include genes associated with other cardiac diseases) to more targeted panels including only genes that are robustly associated with DCM and the patients’ phenotypes. Restricting the analysis to validated and interpretable DCM disease genes (i.e. a core panel of DCM-associated genes) will avoid high numbers of inconclusive, clinically unactionable results.^42^ Broader genetic testing may be appropriate in some situations, particularly for patients who remain gene-elusive after testing the DCM core panel but have a high suspicion of a genetic aetiology of their disease (e.g. in case of multiple affected relatives or DCM at a young age), for patients with overlapping cardiomyopathy phenotypes (e.g. DCM with relative hypertrophy), when there are diverse (cardiomyopathy) phenotypes in one family, or when there is a syndromic form of DCM with additional features (*Figure 2*). Finally, studies have shown a significant increase in genetic yield when genetic testing is re-evaluated or repeated over time when initially no genetic variant was detected.^44^ Besides the medical considerations, it is important to actively involve the patient in selecting the genetic testing strategy. When opting for a broad gene panel, key points to discuss include the risk of uncertain and incidental findings. Possible approaches include an ‘opt-out’ strategy for incidental findings or already discussing options for family segregation analysis should a VUS be identified. Ensuring that patients are well informed about potential outcomes before consenting to genetic testing improves acceptance of the results and enhances the overall impact of genetic testing.

## The interpretation of genetic results in the context of the individual patient

The interpretation of a genetic test result is paramount, as this determines the clinical consequences attributed to the identified variant. Most of the laboratories that perform genetic sequencing will also provide the molecular variant interpretation, as previously described.^40^ In case of a P/LP variant, the treating clinician must interpret the possible causality of the variant to the observed phenotype (i.e. gene–disease relationship) (*Table 3*), in consultation with a clinical geneticist/counsellor, molecular genetic laboratory specialist, appropriately trained cardiologist, or pathologist.

**Table 3:** Cardiac and extracardiac features of common genotypes associated with dilated cardiomyopathy

Gene	Cardiac features^45^	Arrhythmic risk	Extracardiac features
*LMNA*	High prevalence of conduction disorders and atrial and ventricular arrhythmias. Early-onset ventricular dysfunction and poor prognosis	High, *LMNA* risk calculator present: https://lmna-risk-vta.fr/	Skeletal muscle involvement Lipodystrophy
*FLNC*	Cardiac fibrosis and arrhythmias may be the first signs of diseases in the absence of systolic dysfunction	High, FLNC risk calculator present: https://flnctv.shinyapps.io/RiskCalculator/	Skeletal muscle involvement (rare)
*BAG3*	Predominance of HF-associated events	Moderate	
*MYH7*	Presentation at a paediatric age is not uncommon. Prevalent non-compaction phenotypic trait	Low	
*PLN*	Prevalent ventricular arrhythmias and repolarization abnormalities. Low-voltage ECG. Predominant arrhythmic events	High, PLN risk calculator present: https://plnriskcalculator.shinyapps.io/final_shiny/	
*RBM20*	Prevalent ventricular arrhythmias	High	
*TTN*	Can present with acute HF and severe ventricular dysfunction, but good response to HF medication	Low	
*DSP*	Prevalent ventricular arrhythmias and myocardial injury events (recurrent myocarditis)	High, DSP risk calculator present: https://www.dsp-risk.com/	Curly hair and hyperkeratosis of hands and feet

ECG, electrocardiogram; HF, heart failure.

### Interpretation in the context of other contributing factors

Detailed phenotyping of a patient with DCM will help to interpret the causality of a VUS or P/LP variant detected with genetic testing. The probability of a P/LP variant being causative for DCM is high when detected in a proband, justifying clinical consequences. However, when the same variant is found in a different context (e.g. incidental finding and no DCM phenotype), the causal probability is lower and therefore impacts clinical actionability. Additionally, some specific variants are known to be risk factors or low penetrant variants rather than strong causal P/LP variants, requiring other contributing factors to explain the observed phenotype. The disease penetrance of a specific variant (i.e. the chance that a variant may lead to a phenotype) may thus depend on the setting in which the variant is detected.^46^ As an example, some missense variants on specific locations in *LMNA* are associated with a later onset and a milder phenotype with better outcome compared with other P/LP *LMNA* variants.^47,48^ Individuals with *LMNA* variants associated with a milder phenotype have a lower arrhythmogenic risk compared with other *LMNA* variants, and the general *LMNA* risk calculator may therefore be less applicable for these individuals.^49^

### Interpretation in the context of the phenotype of a patient

The patients’ phenotype can contribute to interpreting genetic results in two ways:

1. (Extra-)cardiac features might be present that fit within the clinical spectrum of the affected gene. Examples are skeletal muscle involvement in genes such as *LMNA* and *DES* or the presence of curly hair and hyperkeratosis in patients with P/LP *DSP* variants. Important cardiac genotype–phenotype associations are summarized in *Table 3*. Notably, P/LP variants in *DSP* may manifest with recurrent episodes of chest pain, troponin elevation, and cardiac magnetic resonance (CMR) evidence of myocardial injury, a phenotype often termed ‘*DSP* myocarditis’. These episodes mimic acute viral myocarditis but represent genetically driven myocardial injury with important prognostic, treatment, and arrhythmic implications.^32,50^
2. Genetic results may prompt additional phenotyping (i.e. reverse phenotyping). When broad panels of cardiac-associated genes are used, P/LP variants may be detected in genes that are not (immediately) associated with the patients’ DCM phenotype (e.g. truncating variants in *MYBPC3*). These might either be genetic incidental findings or a genetic clue for the underlying aetiology (i.e. burnt-out hypertrophic cardiomyopathy leading to DCM).^42^

Clinical actionability should be limited to P/LP variants and not to VUSs in genes. Family members should not be tested on VUSs (i.e. cascade screening), unless they have a similar cardiac phenotype. Instead, cardiac screening is recommended for these family members.^4^ Testing affected family members on the VUS (i.e. segregation analysis) can provide additional information necessary to reclassify the variant.

## Clinical consequences of genetic testing in dilated cardiomyopathy

The identification of a genetic aetiology in a patient with DCM has three main clinical consequences: (i) impact on the treatment and follow-up of a patient, (ii) enabling genetic screening and prevention in family members, and (iii) providing reproductive options for the patient. The options may differ depending on the exact gene in which a P/LP variant is detected.

### Impact on treatment and follow-up of a patient

With increasing knowledge of clinical consequences of genetic variants, there is a shift from *disease*-specific towards *gene*-specific risk calculators (e.g. *LMNA*,^49^  *FLNC*^51^, or *DSP*^50^), with emerging efforts even resulting in *variant*-specific risk calculators [e.g. *PLN* p.(Arg14del)].^52^ These calculators can be used to estimate the risk of malignant ventricular arrhythmias and thus be used to stratify patients for primary prevention ICD implantation. Variants in *LMNA*,^53,54^  *FLNC* (truncating variants),^55,56^  *TMEM43*,^57^  *PLN* (p.Arg14del),^52^  *DSP*,^53,54^ and *RBM20*^58,59^ are currently considered as high risk genotypes associated with sudden cardiac death, and ICD implantation should be considered for these patients in the presence of both a LVEF <45% and late gadolinium enhancement (LGE) on CMR according to the 2023 ESC cardiomyopathy guidelines.^2^ In the 2022 ESC guidelines for the management of patients with ventricular arrhythmias and the prevention of sudden cardiac death, the LVEF cut-off value is even on <50%.^60^ For patients with DCM and a P/LP variant in a different gene, ICD implantation may be appropriate in the presence of additional risk factors such as syncope and LGE on CMR.^61,62^

Medical therapy for patients with a genetic form of DCM is not different compared with patients without a genetic substrate, as described in the latest ESC guidelines.^1,2^ However, the individual response to heart failure therapy may be genotype-specific.^53,63,64^ Patients with truncating variants in *TTN* respond on average more favourably to heart failure medication, showing a steep increase in systolic function in the first years following introduction of guideline-directed therapy.^53,65^ However, this therapeutic effect may diminish over time.^66^ For all the other genes, the treatment response is generally lower compared with patients with DCM without a genetic substrate.^53,63,64^

The overall prognosis is worse in patients with DCM with a genetic aetiology; the risk of progression to end-stage heart failure and malignant ventricular arrhythmias is increased.^53^ The clinical course differs depending on the genotype.^53,54^

### Family screening

Family members of a patient with DCM are at increased risk of developing DCM, and for this reason cardiac screening is recommended in first-degree relatives according to the latest 2023 ESC cardiomyopathy guidelines.^2,67^ However, determining the exact risk remains difficult, except if a genetic aetiology has been identified in the family. In this case, family members can be tested for the familial P/LP variant and can be discharged from screening when a P/LP variant is not present. Asymptomatic family members who do carry the P/LP variant should remain under follow-up with cardiac screening every 1–3 years before the age of 60 and every 3–5 years thereafter, according to the latest ESC guidelines on the management of cardiomyopathies.^2^

### Reproductive options

DCM can affect children and (young) adults, many of whom may consider having children in the future. For most genetic forms of DCM, this indicates an a priori risk of 50% that their offspring will inherit the P/LP variant. The finding of a genetic aetiology can provide preimplantation and prenatal testing options that should be discussed with the patient.^68^ Preimplantation genetic testing is a procedure that allows selective implantation of embryos without the P/LP variant.^69^ During pregnancy, prenatal diagnosis via chorionic villus sampling or amniocentesis can be performed to test whether the foetus is affected, although it is not a popular option for inherited cardiac diseases as it can prompt a difficult decision during the pregnancy. When a genetic cause is identified, it is important to assess the patient’s desire to have children to ensure a timely referral for reproductive counselling to provide sufficient time for anticipation and preparation for couples.^70,71^

## Future outlook

Significant improvements in sequencing technologies, in combination with a growing understanding of genetic variation and its clinical consequences, have changed the role of genetics in the diagnosis and management of patients with DCM. Ongoing and future advancements are expected to further strengthen the role of genetics in clinical practice and improve knowledge of genetic or acquired factors that could modulate disease expression, with a more precise gene-tailored approach. International multicentre registries (facilitated by e.g. ERN GUARD-Heart, DCM-SHaRe, and ESC) are indispensable platforms to assess genotype-specific outcomes and long-term therapeutic effects of these rare genetic diseases.

*Gene-specific follow-up and treatment regimens*: the increasing knowledge of genotype–phenotype associations reveals the differences in the natural history and treatment response of genotypes. Although specific genes are already incorporated in decision-making in the 2023 ESC cardiomyopathy guidelines, gene- or even variant-specific recommendations will be an important research area for the near future.^2,45^

*Implementation of polygenic risk scores:* the current diagnostic possibilities are all aimed to find a possible monogenic aetiology of DCM—rare variants with a large effect size. When no genetic aetiology is detected, this does not exclude a potential genetic factor underlying the disease. For example, there could be rare variants that have an additional modest effect size, currently reported as VUS.^42,72^ Additionally, large studies have shown how a combination of single-nucleotide polymorphisms can explain the susceptibility to develop DCM,^17,18^ which will need to be validated in a clinical context and in populations with different ethnicities.

*Artificial intelligence in assisting variant pathogenicity*: the correct interpretation of a genetic variant as either benign or pathogenic is crucial for clinical decision-making. This is currently a very time-consuming and precise task of genetic laboratory specialists. It is likely that artificial intelligence tools will further support the interpretation of genetic variants in clinical practice.^73^

*Novel gene-oriented therapies*: the result of genetics could influence the therapy of a patient in three ways—(i) by guiding the choice of standard heart failure therapy based on gene-specific treatment responses, (ii) through the development of therapies targeting the molecular consequences of a gene defect (e.g. ARRY-371797 in *LMNA*^74^ and danicamtiv in *TTN* and *MYH7*^75^), or (iii) by directly targeting the genetic defect (e.g. gene therapy^76^).

*Training and education in genetics for cardiologists*: the interpretation of genetic results remains the most important step in optimizing the utility of genetic testing. As described in this document, knowledge on the context and genotype–phenotype correlations is essential to form an impactful treatment plan for the patient. Proper education on genetics and genomics for cardiologist is therefore important and could even pave the way for genetic cardiologists as sub-specialism.^77^

## Summary

In this clinical consensus statement, we highlight the current knowledge on the genetic aetiology of DCM and how to implement genetic testing in routine clinical practice of care for patients with DCM. Knowledge of the genetic aetiology and genotype–phenotype associations remains important for interpreting genetic testing results in a meaningful way to guide clinical decision-making and recommendations for family members. Education of cardiologists in genetics and genomics, along with the possibility to discuss cases with specialized centres, is essential to further expand the role of genetics in patient care.

## Declarations

### Data Availability

No data were generated or analysed for this manuscript.
